# PCSK9 inhibition: the way forward in the treatment of dyslipidemia

**DOI:** 10.1186/s12916-015-0503-4

**Published:** 2015-10-12

**Authors:** Robert M. Stoekenbroek, John JP Kastelein, Roeland Huijgen

**Affiliations:** Department of Vascular Medicine, Academic Medical Center, University of Amsterdam, PO Box 22660, Meibergdreef 9, 1105 AZ Amsterdam, Netherlands

**Keywords:** Cholesterol-lowering drugs, LDL-cholesterol, PCSK9

## Abstract

Barely a decade after the discovery of the gene encoding proprotein convertase subtilisin/kexin type 9 (PCSK9) and its recognition as a key player in cholesterol metabolism, PCSK9 inhibition is now considered an exciting approach in the reduction of residual risk of cardiovascular disease. The progress from PCSK9 discovery to the development of targeted treatment has been unprecedented in terms of scale and speed. The first suggestion of a link between PCSK9 and hypercholesterolemia was published in 2003; a decade later, two meta-analyses of clinical trials comparing anti-PCSK9 treatment to placebo or ezetimibe, including >10,000 hypercholesterolemic individuals, were published. Currently, three PCSK9 inhibitors are being evaluated in clinical outcome trials and the results will determine the future of these lipid-lowering therapies by establishing their clinical efficacy in terms of cardiovascular event reduction, safety, and the consequences of prolonged exposure to very low levels of LDL-cholesterol. Irrespective of their outcomes, the exceptionally rapid development of these drugs exemplifies how novel technologies, genetic validation, and rapid clinical progression provide the tools to expedite the development of new drugs.

## Background

Two decades after the results of the Scandinavian Simvastatin Survival Study first showed that statins effectively improve survival in patients with cardiovascular disease (CVD), thus initiating a revolution in the treatment of dyslipidemia [1], ezetimibe has been the only drug shown to further improve outcomes for dyslipidemic patients [2]. Research programs of novel compounds were prematurely halted due to safety concerns or a lack of efficacy and the use of such drugs was often associated with only modest reductions in low-density lipoprotein cholesterol (LDL-C) [3–6]. Established lipid-modifying compounds, such as fibrates and nicotinic acids, failed to improve CVD events as the primary outcome when used in conjunction with optimal statin therapy [7, 8]. Therefore, recent guidelines no longer recommend the routine use of non-statin drugs in conjunction with high-intensity statin treatment [9]. Nevertheless, many patients fail to achieve acceptable lipid control with or are unable to tolerate statin treatment [10, 11]. Additionally, consideration of the potential merits of further LDL-C reductions resurfaced when the results of the IMProved Reduction of Outcomes: Vytorin Efficacy International Trial (IMPROVE-IT) study indicated that addition of ezetimibe to simvastatin significantly reduced the risk of subsequent CVD events in patients with acute coronary syndromes [2]. Given these considerations and since the discovery of the association between mutations in proprotein convertase subtilisin/kexin type 9 (PCSK9) and familial hypercholesterolemia (FH) in 2003 [12], PCSK9 inhibitors have emerged as the prime candidate to further improve outcomes for CVD patients and may initiate the next revolution in anti-atherosclerotic therapy.

## Discovery and function

**Fig. 1:**
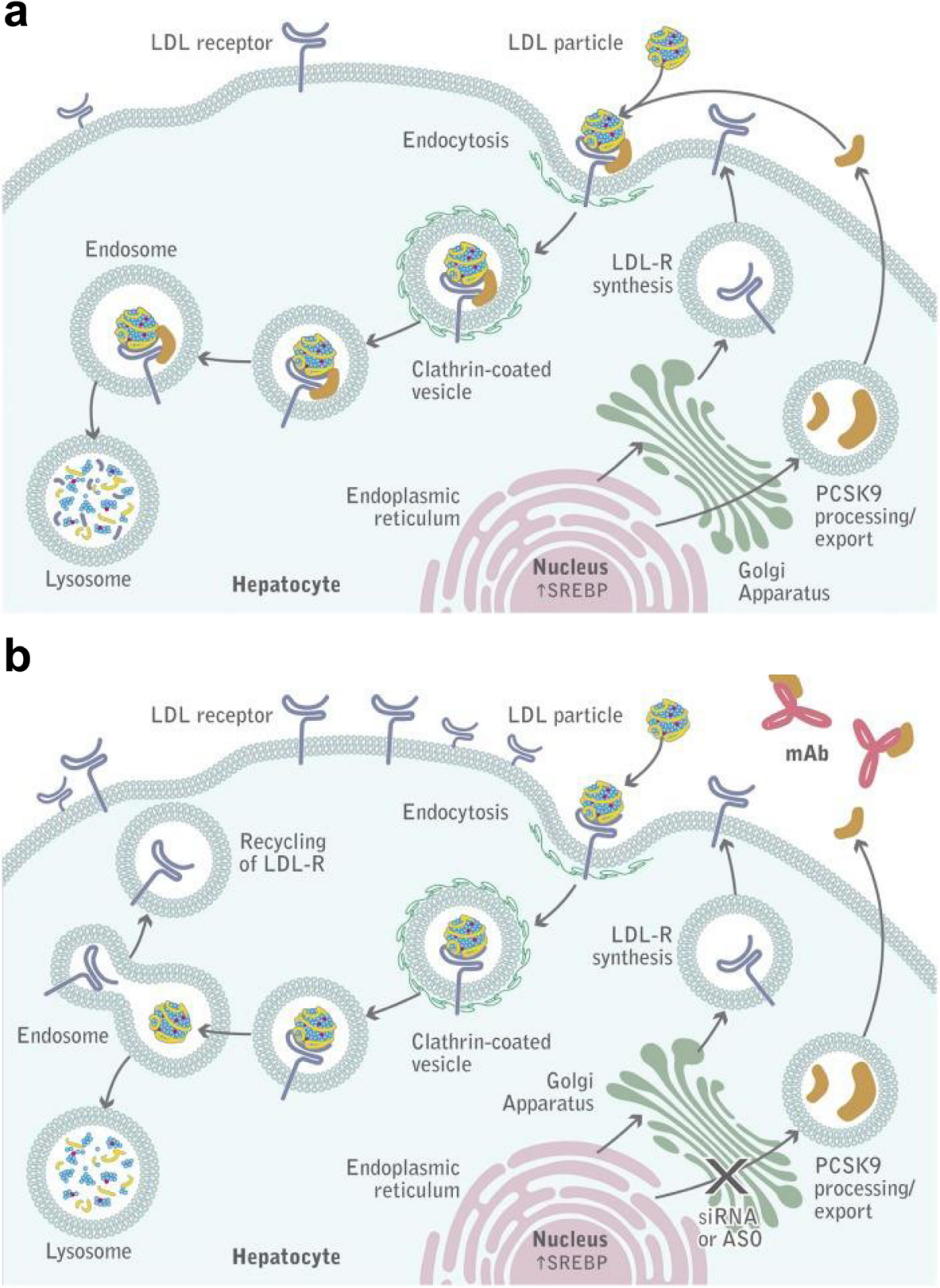
**a**. PCSK9-mediated degradation of low-density lipoprotein receptor (LDLR). A complex of low-density lipoprotein cholesterol (LDL-C), LDLR, and PCSK9 is internalized into hepatocytes within clathrin-coated pits and subsequently undergoes lysosomal degradation. **b**. PCSK9 inhibition. Monoclonal antibodies bound to PCSK9 prevent the association between PCSK9 and LDLR. LDLR binds and internalizes LDL-C particles, which are then degraded in the lysosome, whereas the LDLR is recycled back to the plasma membrane. Re-use kindly permitted by the American Society for Biochemistry and Molecular Biology, Inc. Figure derived from The PCSK9 decade [13]

PCSK9 was recognized to play an important role in LDL-C metabolism after the identification of gain-of-function mutations in two French families with FH without mutations in other FH-associated genes [12]. Subsequent experiments revealed that PCSK9 increases levels of LDL-C by reducing the available pool of hepatic LDL-receptors [13]. In the absence of PCSK9, the LDL-receptor is recycled back to the plasma membrane. Binding of PCSK9, on the other hand, prevents LDL-receptor recycling and instead targets it for lysosomal degradation (Fig. 1a) [13]. An extensive review of the underlying mechanisms of PCSK9 inhibition was recently provided by Lambert et al. [13]. Large cohort studies have revealed associations between variations in the PCSK9 gene and LDL-C levels and CVD risk [14]. In addition, studies have shown that statin treatment increases PCSK9 levels [13]. The inverse relation between PCSK9 activity levels and LDL-receptors suggests that PCSK9 inhibition could have a synergistic effect with statins on LDL-C. Therefore, PCSK9 has been identified as a promising target for anti-atherosclerotic drug development. Several strategies have been developed to reduce PCSK9 function, including binding of plasma PCSK9 by monoclonal antibodies, reducing PCSK9 expression by silencing RNA, or vaccination against PCSK9 [15, 16]. The present review focuses primarily on PCSK9-inhibiting antibodies due to their presently more advanced clinical development.

## Development of PCSK9-inhibiting antibodies

The first reports of phase I studies of three PCSK9-inhibiting antibodies, the mechanism of action of which is shown in Fig. 1b, were published a mere decade after the discovery of PCSK9. In their landmark study, Stein et al. [17] demonstrated the ability of the PCSK9-inhibiting antibody alirocumab to reduce LDL-C by more than 60 % in both healthy volunteers and patients with FH. Subsequent phase I studies of other PCSK9 inhibitors, including evolocumab and bococizumab, confirmed these findings [18]. Hitherto, the clinical potential of PCSK9 inhibition with monoclonal antibodies has been studied in more than 20 short-term clinical trials against various background lipid-lowering therapies and with both placebo and ezetimibe as a comparator treatment. The populations reflected the major categories of patients for whom PCSK9 inhibition will likely be used in practice: familial- and non-familial hypercholesterolemia, statin-intolerant patients, and high-risk secondary prevention patients who fail to achieve acceptable lipid goals with currently available treatment options.

## Effect of PCSK9-inhibiting antibodies

Two recent meta-analyses evaluated the clinical potential of antibody PCSK9 inhibition in >10,000 patients who participated in over 20 phase II and III trials [19, 20]. These meta-analyses included data from most of the available studies with alirocumab and evolocumab. Not included in the first meta-analysis were observations from the Open-Label Study of Long-Term Evaluation against LDL Cholesterol (OSLER), which was an open label extension study of participants who had completed phase II or III studies (“parent trials”) of evolocumab [21]. The results were, in essence, similar to those from the large dataset in the meta-analyses [19]. In contrast to the substantial body of evidence on alirocumab and evolocumab, longer-term safety and efficacy data on bococizumab is relatively limited, with only one full publication on 351 patients available to date [22].

## Effects on lipids

All trials confirmed the beneficial effect of PCSK9 inhibition with evolocumab and alirocumab on LDL-C levels. The use of PCSK9 monoclonal antibodies was associated with mean LDL-C reductions of approximately 50 % [19, 20]. These results persisted during follow-up, and were similar across different doses, patient populations, and in patients with or without background statin therapy [19–21]. Compared to ezetimibe, PCSK9 inhibition resulted in a mean LDL-C reduction of approximately 36 % (95 % confidence interval (CI), 33–39 %) [19]. In addition to their LDL-C lowering effect, PCSK9 inhibitors consistently increased high-density lipoprotein cholesterol by approximately 6 % (95 % CI, 6–7 %) and lowered lipoprotein(a) by approximately 26 % (95 % CI, 23–30 %) both in placebo- and ezetimibe-controlled trials [19]. The effects on lipid levels were similar in trials on evolocumab, alirocumab, and bococizumab [19, 20, 22].

## Clinical efficacy

The published individual trials were not powered to study the efficacy in terms of clinical outcomes. In total, 10 randomized controlled trials, with over 5000 participants, reported data on myocardial infarction [19]. One of the meta-analyses studied myocardial infarction rates and showed a significantly lower rate (0.6 %; 19/3289 patients) in PCSK9 inhibitor-treated patients compared to those who received no anti-PCSK9 treatment (1.0 %; 19/1906 patients; odds ratio, 0.49; 95 % CI, 0.26–0.93) [19]. Similarly, statistically significant reductions in CVD event rates were reported during the 1-year follow-up of the OSLER and ODYSSEY long-term trials [21, 23]. Furthermore, these preliminary analyses suggest a statistically significant reduction in all-cause mortality and a non-significant reduction in CVD mortality. Because of the limited number of events, long-term clinical outcome studies should be awaited to substantiate these findings.

## Safety

Pooled analysis of serious adverse event rates from phase II and III studies demonstrated similar incidence rates in patients treated with PCSK9 inhibitors versus controls [19]. Similarly, there were no differences in the incidence of any adverse events compared to placebo, irrespective of the dosage of evolocumab [20]. Moreover, adherence rates were similar in both groups. Given that PCSK9 inhibitors could be a valuable option for statin-intolerant patients, the observation that elevated creatine kinase occurred less frequently in PCSK9 inhibitor-treated patients than in controls is of interest [19, 20]. None of the phase III trials reported the presence of neutralizing antibodies. The favorable safety profile of PCSK9 inhibitors [24], as well as the results of several meta-analyses of statin trials [25], provide reassuring evidence on the short-term safety of achieving very low levels of LDL-C. The observation of a slightly increased occurrence of neurocognitive dysfunction in patients receiving alirocumab or evolocumab in the ODYSSEY long-term trial (1.2 % vs. 0.5 %; *P* = 0.17) [21] and OSLER trial (0.9 % vs. 0.2 %; *P* = 0.3) [23] has led to the initiation of a study designed specifically to evaluate neurocognitive effects of PCSK9 inhibition with evolocumab in 4000 individuals (NCT Trial Identifier: NCT02207634). Further, inherent to monoclonal antibodies is their subcutaneous administration. A prevalence of approximately 5 % of injection site reactions was reported by patients in the active treatment arms with evolocumab or alirocumab [21, 23].

## Future perspectives

The results of large ongoing clinical outcome trials of PCSK9 inhibitors will determine the way forward in the treatment of dyslipidemia since these trials will indicate whether additional LDL-C lowering safely translates into cardiovascular benefit. Although it would be premature to endorse these drugs for widespread use before the results of ongoing trials are available, it is interesting to speculate on the categories of patients for whom PCSK9 inhibition would be first applied. Notably, the United States Food and Drug Administration has recently approved alirocumab, in addition to diet and maximally tolerated statins, in patients with heterozygous FH or clinical CVD who require additional LDL-C lowering [26]. Similarly, the European Medicines Agency has recommended approval of alirocumab and evolocumab for patients who fail to achieve acceptable lipid control despite optimal statin therapy, but also explicitly mentions patients with homozygous FH and statin-intolerant patients [27, 28]. Arguably, the largest group of patients who will potentially benefit from PCSK9 inhibition consists of those remaining at increased risk of CVD despite currently available lipid-lowering treatment due to, for example, having a very high baseline CVD risk. Statin treatment will likely remain the basis of lipid lowering treatment in the foreseeable future due to their relatively low costs, oral availability, and their established beneficial safety profile and clinical efficacy. However, results of the IMPROVE-IT study confirmed the ‘lower is better’ paradigm regarding LDL-C levels and CVD risk, demonstrating additional clinical benefit through the addition of ezetimibe to simvastatin [2]. Importantly, the risk reduction associated with additional LDL-C lowering appears to be independent of baseline LDL-C [2, 24]. Discussion on the potential role of PCSK9 inhibitors in the treatment of this large and heterogeneous group of patients will therefore focus on the desired balance between additional absolute risk reduction and costs. In the IMPROVE-IT trial, addition of ezetimibe to simvastatin resulted in an absolute risk reduction of 2 % in CVD events after 7 years of treatment, or a number needed to treat for 5 years of approximately 70 to prevent one CVD event [2]. The number needed to treat for the more potent PCSK9 inhibitors will likely be three-fold lower [21, 23].

Another group of patients who may benefit from PCSK9 inhibitors could be those with a strict indication for lipid lowering but who are statin intolerant. Indeed, this category of patients is included in the recent European Medicines Agency recommendation [27]. Although multiple definitions of statin intolerance exist, the magnitude of the problem is illustrated by the observation that 75 % of patients discontinue statins within 2 years, with statin-associated muscle symptoms being the prevailing reason in approximately 60 % of cases [10, 29]. Evolocumab and alirocumab showed a good safety and tolerability profile in statin-intolerant patients [30–32]; bococizumab is being tested in statin-intolerant patients (NCT Trial Identifier: NCT02135029). The potential of PCSK9 inhibitors as an alternative for statin-intolerant patients who fail to achieve acceptable LDL-C control on currently available therapies has been recognized in the current European Atherosclerosis Society guideline [33].

Furthermore, PCSK9 inhibition may be of benefit in the treatment of patients with homozygous FH. Currently available oral lipid-lowering agents do lower LDL-C in most homozygous FH patients, but the limited efficacy means that these patients are currently often dependent on invasive treatment modalities such as LDL-C apheresis or Apolipoprotein B or microsomal triglyceride transfer protein inhibition, with the latter two having been associated with steatosis [34–37]. Evolocumab has been proven efficacious in reducing LDL-C in a 12-week proof of concept, randomized controlled trial during including 50 homozygous FH patients, but only in those carrying alleles of the LDL-receptor with some residual activity, as can be expected from the mechanism of action of PCSK9 inhibition (Fig. 1) [38]. Similarly, despite the potency of statins, only a minority of patients with heterozygous FH attain optimal cholesterol levels and considerable excess mortality still exists [11, 39]. Published trials of PCSK9 inhibitors have already demonstrated the potential to reach unprecedented low LDL-C levels in heterozygous FH and currently ongoing clinical outcome studies will establish whether addition of PCSK9 inhibition to current treatment confers sustained clinical benefit in this patient population (NCT Trial Identifier: NCT01968980) [40–42].

All of the four ongoing clinical outcome trials on PCSK9-inhibition antibodies include high-risk patients (either clinically manifest CVD or dyslipidemia) and will determine the efficacy of PCSK9 inhibitors added to lipid-lowering therapy in reducing major CVD event rates compared to placebo over approximately 5 years of follow-up (Table 1). The first results of these trials are expected in 2018. Ultimately, the societal benefit of these treatments will depend on their costs, safety, and efficacy. The expected high cost of PCSK9 inhibitors could indicate that their use may only be cost-effective in the treatment of patients with a particularly high baseline risk; a crucial challenge will be defining the level of baseline risk at which PCSK9 inhibition is valuable. The benefit of PCSK9 inhibition in practice will further depend on therapy compliance and therefore dose and frequency of administration need to be carefully considered. Finally, despite the fact that ongoing phase III trials will determine the safety of PCSK9 inhibition after 5 years of follow-up, post-marketing registration and extended follow-up studies are required to confirm their safety in the longer term.

**Table 1:** Ongoing clinical outcome trials of PCSK9 inhibitors

Trial name	Study drug	Patient population	Primary outcome measure	Follow-up
FOURIER NCT01764633	Evolocumab	*n* = 27,000; history of CVD – at high risk of recurrent event; LDL-C ≥70 mg/dL or non-HDL-C ≥100 mg/dL; background statin therapy	Time to cardiovascular death, myocardial infarction, hospitalization for unstable angina, stroke, or coronary revascularization	5 years
ODYSSEY OUTCOMES NCT01663402	Alirocumab	*n* = 18,000; acute coronary syndrome <52 weeks earlier; LDL-C ≥70 mg/dL or non-HDL-C ≥100 mg/dL; background statin therapy	Time to cardiovascular death, non-fatal myocardial infarction, hospitalization for unstable angina, or stroke	64 months
SPIRE-1 NCT01975376	Bococizumab	*n* = 17,000; high risk of CVD event, primary and secondary prevention; background lipid-lowering treatment; LDL-C 70–100 mg/dL or non-HDL-C 100–130 mg/dL	Time to composite major cardiovascular event (cardiovascular death, non-fatal myocardial infarction, non-fatal stroke, and hospitalization for unstable angina)	60 months
SPIRE-2 NCT01975389	Bococizumab	*n* = 9000; high risk of CVD event; background lipid-lowering treatment; LDL-C ≥100 mg/dL or non-HDL-C ≥130 mg/dL	Time to composite major cardiovascular event (cardiovascular death, non-fatal myocardial infarction, non-fatal stroke, and hospitalization for unstable angina)	60 months

*CVD* Cardiovascular disease, *HDL-C* High-density lipoprotein cholesterol, *LDL-C* Low-density lipoprotein cholesterol

## Conclusions

Merely a decade after the discovery of the essential role of PCSK9 in LDL-C metabolism, its inhibition has emerged as one of the most promising novel strategies to reduce CVD. Their remarkable efficacy in reducing LDL-C and the possible synergistic effects with statins, combined with a favorable safety profile and tolerability, provide these drugs with the potential to revolutionize the treatment of patients at high risk of CVD. Ongoing clinical outcome trials will provide a definite answer to the question on the clinical benefit of further reducing LDL-C. Irrespective of their results, the rapid development of these drugs illustrates the possibilities offered by new technologies and genetic research methods.

## Abbreviations

CI: Confidence interval
CVD: Cardiovascular disease
FH: Familial hypercholesterolemia
IMPROVE-IT: IMProved Reduction of Outcomes: Vytorin Efficacy International trial
LDL-C: Low-density lipoprotein cholesterol
OSLER: Open-Label Study of Long-Term Evaluation against LDL Cholesterol
PCSK9: Proprotein convertase subtilisin/kexin type 9
